# Endometriosis and ovulatory menstruation: beyond the Sampson principle

**DOI:** 10.1172/JCI188787

**Published:** 2025-07-01

**Authors:** Serdar E. Bulun

**Affiliations:** Department of Obstetrics and Gynecology, Northwestern University, Chicago, Illinois, USA.

## Abstract

Endometriosis is an estrogen-dependent chronic inflammatory syndrome characterized by viable endometrial tissue outside the uterine cavity and associated with pain and infertility. Endometriosis, as tissue or a pathological process, is dynamic in that its establishment and progression require repeated episodes of retrograde travel of shed endometrial tissue, which implants in the lower abdominal cavity following ovulatory cycles and survives. Estrogen-rich follicular fluid released onto peritoneal surfaces during ovulation may also support endometriotic implants. DNA evidence indicates that endometriosis originates from eutopic endometrial tissue, which may reach the abdominal cavity in a retrograde manner primarily via the uterine tubes. Unlike uterine bleeding associated with non-ovulatory circumstances, retrograde menstruation following an ovulation maximizes shedding of epithelial cells localized to deep invaginations of the basalis portion of the endometrium, which likely carry somatic cancer-driver mutations such as KRAS. The attached endometrial stromal cells are mostly mutation free but display epigenetic defects including overexpression of aromatase and estrogen receptor-β and downregulation of progesterone receptor, causing estrogen excess and progesterone resistance. These tissue clones may form implants in involuting ovarian corpus luteum cysts and peritoneal surfaces and induce tissue remodeling and fibrosis, manifested as deep-infiltrating endometriosis. The first-line treatment for chronic pelvic pain associated with endometriosis is suppression of ovulation, with the goal of relieving pain. Infertility is often managed using in vitro fertilization, which improves the embryo quality and alters endometrial development.

## Clinical features

**Figure 1:**
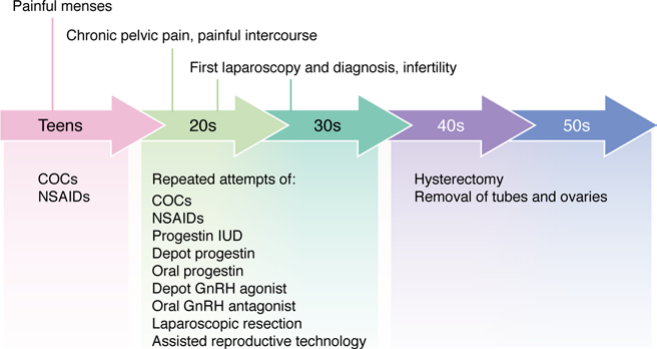
The clinical course of endometriosis over a lifetime. The top row summarizes typical patient experiences beginning in the teenage years and continuing through their 40s and 50s. The lower row lists pain management strategies recommended during each decade of life. Although hysterectomy and bilateral removal of tubes and ovaries are frequently used, they represent severely radical approaches and may not provide pain relief in a subset of patients. COCs, combined oral contraceptives; NSAID, nonsteroidal antiinflammatory drug; GnRH, gonadotropin-releasing hormone.

This Review focuses on the role of the menstrual event that follows ovulation in the pathophysiology of endometriosis. Endometriosis is a unique inflammatory syndrome resulting from repeated episodes of ovulatory menstrual bleeding in a subset of people who menstruate, a population comprising diverse gender and biological sex identifications. This Review will mainly refer to women because they are highly represented in the clinical literature (1, 2). Endometriosis, as tissue or a pathological process, is not static but dynamic in that repeated episodes of retrograde travel of shed endometrial tissue, which implants and survives in the lower abdominal cavity following ovulatory cycles, are indispensable for its establishment and progression (1, 2). Its primary symptoms are painful menstrual periods, chronic pelvic pain, painful intercourse, and infertility (3–6). The dependence of endometriosis on estrogen to drive the inflammatory process and pain separates it from other chronic inflammatory syndromes such as Crohn’s disease or acid reflux esophagitis (7, 8). Until recently, a prevailing notion that prioritized the surgical description of endometriosis over the disease process unfortunately limited this syndrome to a narrow anatomic definition, i.e., the ectopic presence of endometrium-like tissue outside of the uterine cavity; this definition profoundly stalled progress for developing long-term and patient-centered management strategies (9, 10). This arbitrary weighted and purely anatomic classification system had limited value to predict relief of pain or achievement of pregnancy in response to various regimens of symptom management (11, 12). A recent shift toward developing new paradigms to understand and manage the symptoms of endometriosis through the various stages of the disease process puts patients first (4, 6, 13, 14). This new overall approach recognizes its early manifestations during adolescence, the long history of disease, its recurrent symptoms, and the overall limitations of the currently available medical and surgical approaches (Figure 1).

**Figure 2:**
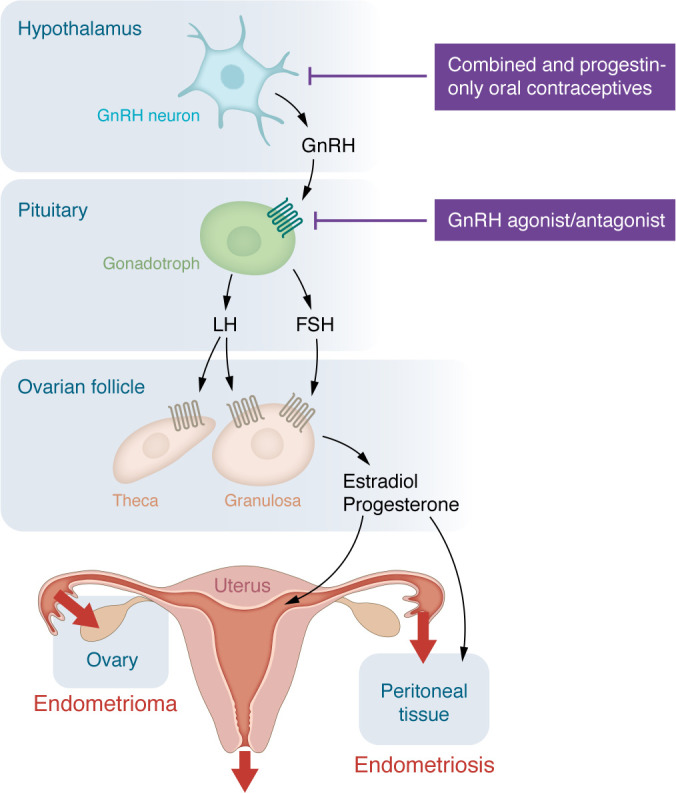
Ovulatory cycle. Repetitious ovulatory cycles initiated by hypothalamic gonadotropin-releasing hormone (GnRH) neurons are essential for the establishment and progression of endometriosis (see Figure 3). GnRH, which is secreted in a pulsatile manner, acts on its seven-transmembrane G protein–coupled receptor on the gonadotroph and induces sequential secretion of follicle-stimulating hormone (FSH) and luteinizing hormone (LH) from the pituitary. FSH and LH, via their specific G protein–coupled receptors on ovarian granulosa and theca cells, stimulate sequential secretion of estradiol and progesterone. Estradiol during the proliferative phase builds the functionalis layer of the endometrium, whereas progesterone during the secretory phase stimulates endometrial differentiation. In the absence of pregnancy, endometrial tissue is shed through the vagina and also in a retrograde manner through the uterine tubes into the lower abdominal cavity.

A number of hypotheses have been proposed to explain the presence of endometrial tissue outside the uterine cavity (15). This Review focuses on the mechanism, which is supported by an extraordinarily large body of consistent scientific evidence that indicates endometriosis and its symptoms are caused by retrograde travel of menstrual blood containing molecularly defective endometrial tissue fragments onto the lower abdominal peritoneal surfaces and the ovaries following an ovulation (Figure 2) (16–19). In 1927, John Sampson was the first to provide evidence that this retrograde spread into the pelvic cavity could take place via the uterine tubes or venous circulation (18–20). Also known as Sampson’s theory, this straightforward mechanism was recently demonstrated via mapping of the identical DNA variants or mutations in the epithelial cells of the endometrium to the ectopic endometriotic implants in hundreds of endometriosis patients (21–25). Adenomyosis, a disorder defined as the presence of multiple islands of endometrial tissue in the myometrium, is closely related to endometriosis (26, 27). Adenomyosis results from survival and growth of entrapped fragments of basalis endometrium in the myometrial layer (25, 27). This concept of intracavitary endometrium as the source of pelvic endometriosis and its symptoms has also been emphasized in a number of recent review articles (4, 6, 14).

There is also consensus that endometriosis likely affects a woman chronically from adolescence until menopause (Figure 1) (4, 14). The paradigm that dysmenorrhea should be defined as primary or secondary (secondary being associated with pelvic pathology) is unhelpful and implies that imaging or laparoscopy is necessary to make a presumptive diagnosis of endometriosis (28). Labeling severe menstrual cramps as primary dysmenorrhea during adolescence just because the patient has not gone through a workup including laparoscopy assumes, by definition, that dysmenorrhea can be managed without understanding of its etiology if ovulation is suppressed together with regimens of NSAIDs for an undefined period of time (28, 29). This diagnostic approach does not allow the clinician to consider long-term preventative measures for the progress of endometriosis. Moderate to severe dysmenorrhea experienced during adolescence likely represents the initial stages of the long disease process of endometriosis (Figure 1). The modern, patient-centric definition of endometriosis is more clinically helpful, since it is used to describe the symptom complex that a patient experiences throughout their reproductive life (Figure 1) (4, 6, 14). An endometriosis patient may or may not have the surgically demonstrable presence of endometrium-like tissue in their lower abdominal organs, including the ovaries, because microscopic disease may be missed. In approximately 10% of patients endometriosis and its symptoms persist after menopause or even after the surgical removal of their uterus and ovaries (8, 30, 31).

### Epidemiology, symptoms, and clinical course

Endometriosis is the most common cause of painful menses and chronic pelvic pain in women; and it is often associated with infertility (5, 32, 33). Worldwide, approximately 200 million reproductive-age women suffer from endometriosis and experience painful and heavy menstrual bleeding, painful intercourse, and pelvic pain outside of menses (34, 35). More than 100,000 hysterectomies and many more laparoscopies for the diagnosis and conservative removal or ablation of endometriosis are performed annually in the United States (36, 37). Multiple studies showed that the suffering and economic burden associated with endometriosis are very high and resemble those of other chronic inflammatory conditions such as Crohn’s disease and rheumatoid arthritis, except that endometriosis may be more prevalent (38, 39). The analyses used in these studies focus on health care costs and usually severely underestimate productivity loss and decreased quality of life (38, 39). There are additional obstetrical risks associated with endometriosis: a history of endometriosis increases the risk of early pregnancy loss, preterm delivery, neonatal admission to the intensive care unit, and gestational diabetes (40, 41).

Diffuse adenomyosis is associated with prolonged menstrual bleeding, painful menses, and recurrent pregnancy loss (26, 27, 42). The presence of adenomyosis together with endometriosis elevates the risk of placenta previa or a cesarean delivery (40).

The hallmark symptoms of endometriosis, i.e., dysmenorrhea, dyspareunia, and chronic pelvic pain, are associated with retrograde travel of menstrual blood and tissue and an inflammatory response (Figure 2) (1, 34, 43–46). Stimulation of the nociceptors on peritoneal surfaces by the menstrual tissue or established ectopic implants is thought to be the major source of pain (47, 48). Nerve-like tissue has been described in the endometrium of patients with endometriosis, raising the question of whether intracavitary endometrium may also contribute to the generation of pain (49–51). Additionally, nociceptive input from the spinal cord to higher brain areas may amplify receptor activation at peripheral tissues (47).

In the majority of patients, endometriosis first manifests as moderate to severe adolescent dysmenorrhea, which either goes untreated or is managed with NSAIDs or combination oral contraceptives (Figure 1) (52). Many of these patients eventually develop persistent pain between their periods and experience painful intercourse (53). Only a fraction of these patients undergo laparoscopy that may reveal pelvic endometriotic implants and facilitate their surgical removal to provide temporary pain relief (Figure 1) (53, 54). Pain recurs in up to 90% of these patients within 2 years despite adjuvant ovarian suppression; this eventually leads to repeated laparoscopic procedures in an attempt at pain control (Figure 1) (54). This population of patients with relapsed symptoms constitutes the largest and the most challenging group among all patients with endometriosis, since after each treatment attempt the recurrent symptoms progressively become harder to manage (54). Many of these patients eventually resort to surgical removal of the uterus and the ovaries (Figure 1) (31). Even these radical approaches do not provide pain relief in at least 10% of patients (Figure 1) (8, 30, 31).

As adjuvant approaches to surgical resection, suppression of ovulation or menses for pain control is attempted by the use of progestin-containing intrauterine devices, combination oral contraceptives, or gonadotropin-releasing hormone (GnRH) analogs before or after surgical procedures (55). Given that the majority of cases of endometriosis occur as a result of retrograde travel of endometrial tissue to the abdominal cavity, it is my opinion that long-term suppression of ovulatory menses should be the mainstay of prevention or therapy. Surprisingly, this approach as the primary treatment of endometriosis is seldom considered, recommended, or used (2, 56).

### Role of ovulatory menses in endometriosis

Ovulatory menstruation per se is essential for the development of symptomatic endometriosis (Figure 2) (2, 14, 56). Only menstruating species develop endometriosis (2, 56–59). Women with conditions precluding spontaneous menstruation, such as Müllerian aplasia or Turner syndrome (caused by loss or partial loss of an X chromosome), do not develop endometriosis, although the presence of a vestigial Müllerian remnant has been published in a rare patient with Turner syndrome (60). Endometriosis and anovulatory disorders such as polycystic ovary syndrome rarely occur together (61, 62).

**Figure 3:**
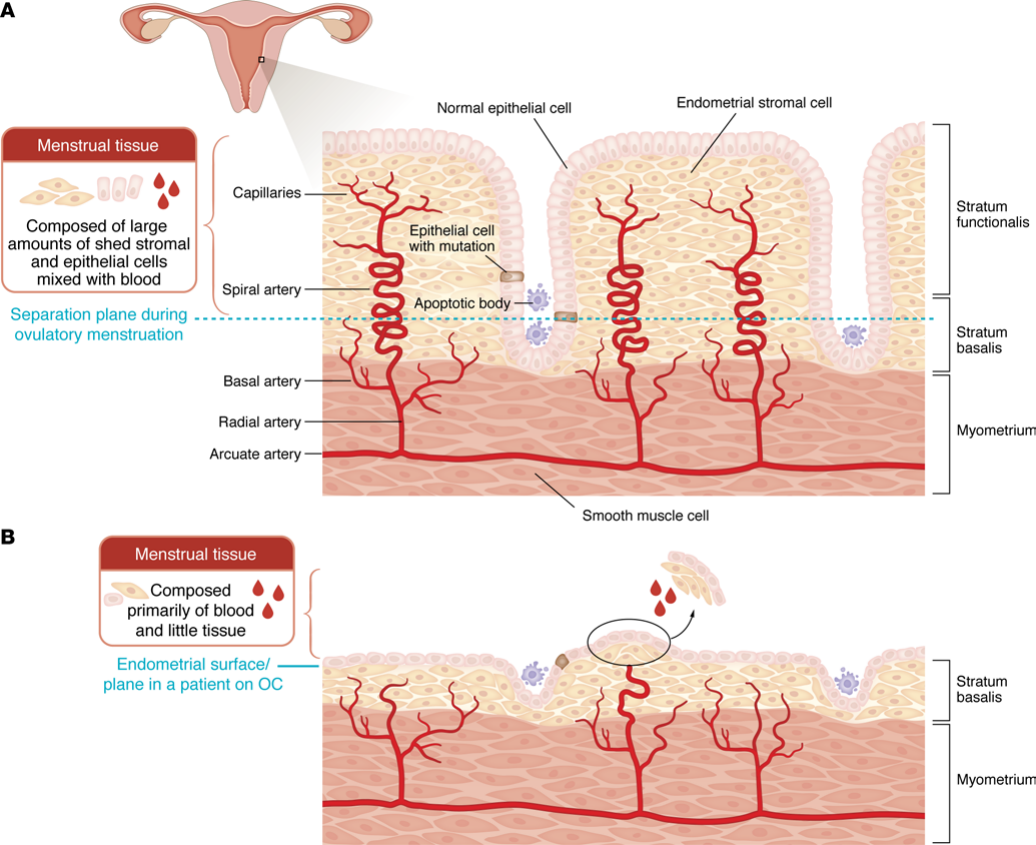
Not all forms of uterine bleeding increase the risk of endometriosis. (**A**) Menstrual tissue after a nonpregnant ovulatory cycle. In addition to inducing stromal and epithelial differentiation, progesterone during the secretory phase of the menstrual cycle stimulates the coiling of the spiral arteries (64, 65). When progesterone production is interrupted in a nonpregnant cycle, the spiral arteries of the basalis and functionalis layers go through rapid and intense vasoconstriction and coagulation, leading to the separation of the thick functionalis layer in its entirety. This leads to the presence of large numbers of viable stromal, epithelial, and endothelial cells along with blood in the menstrual tissue. Some of these tissue fragments travel through the tubes into the pelvic cavity. During this process, mutated epithelial cells and epigenetically abnormal populations of stromal cells are deposited on the pelvic peritoneum or the ovaries. (**B**) Menstrual tissue associated with withdrawal bleeding in a patient on an oral contraceptive (OC). Because the endometrial tissue that is chronically exposed to an estrogen-progestin combination or progestin-only OC does not build a thick functionalis layer, temporary cessation of OC gives rise to menstrual tissue composed primarily of blood and containing very few stromal or epithelial cells (65, 76). This is also the case for the breakthrough bleeding the patient experiences while they are on hormone-containing OC pills used in a continuous manner.

Repetitious menstrual episodes that follow ovulation represent the single most impactful and indispensable factor that initiates the process of endometriosis and worsens its existing symptoms, for the following reasons (Figure 2): First, during an ovulatory cycle, sequential exposure to estradiol for 2 weeks followed by added secretion of progesterone for another 2 weeks maximizes endometrial thickness right before this large mass of tissue is shed (Figure 3A) (63–65). Physiologically developed spiral arterioles and endometrial vessels during this period may lead to rapid vasoconstriction and separation at a plane close to the myometrium, producing large quantities of viable endometrial tissue available for transtubal migration onto the pelvic peritoneal surfaces and ovaries (66). Thus, large quantities of viable endometrial tissue are likely shed after a nonpregnant ovulatory cycle, and some of this tissue together with blood reaches into the lower abdominal cavity via the tubes (Figure 3A). Second, massive quantities of estradiol (as high as 130,000 pmol/L in the follicular fluid of approximately 5 mL) and pain-inducing substances such as prostaglandin E_2_ are released directly onto the pelvic peritoneal surfaces during an ovulation (67, 68). These strikingly high quantities of estradiol probably contribute to the implantation and growth of viable endometrial fragments on peritoneal or ovarian surfaces. Third, an ovarian endometrioma may be formed in a hemorrhagic corpus luteum cyst that recently released an egg (Figure 2) (69). The steroid hormone–rich cyst cavity serves as a growth medium for endometrial tissue to survive and thrive. Fourth, compared with women having regular predictable periods, the prevalence of endometriosis is lower in anovulatory states such as polycystic ovary syndrome, hypothalamic amenorrhea, or breastfeeding (61, 70). Fifth, early menarche followed by cyclic periods is associated with a higher risk of endometriosis (2, 71). Sixth, during infertility treatment of a patient with endometriosis, back-to-back cycles of ovulation induction followed by intrauterine insemination worsen endometriosis symptoms (72), possibly because these simple but low-fecundity treatments must be repeated multiple times, exposing the patient to multiple ovulatory menstrual cycles. On the other hand, more intense forms of ovulation induction used for in vitro fertilization (IVF) usually do not worsen endometriosis as much as intrauterine induction cycles, possibly because although IVF is associated with very high levels of estradiol, it does not trigger multiple and frequent episodes of ovulatory menstruation (72).

Additionally, time-honored clinical data support the postulate that repetitious menstrual episodes that follow ovulation contribute to the development or worsening of endometriosis symptoms. For example, the conventional use of cyclic combination oral contraceptive pills involving withdrawal bleeding is associated with cyclic uterine bleeding that is much lighter than spontaneous ovulatory menses (Figure 3B) (65, 73–76). This may be because a more superficial and thinner portion of endometrial tissue is present in the menstrual tissue (Figure 3B). Oral contraceptive use, which disrupts or suppresses ovulatory menses, reduces endometriosis symptoms such as dysmenorrhea or chronic pelvic pain (74). Breakthrough bleeding associated with other anovulatory conditions such as polycystic ovary syndrome or postmenopausal hormone replacement does not increase the risk of endometriosis (61, 70). It follows that suppression of ovulation should be pursued as a first-line approach for preventing the development of endometriosis or alleviating its symptoms (77).

## Key mechanisms of endometriosis

The fundamental mechanism of endometriosis is relatively straightforward. Following each nonpregnant ovulatory cycle, menstruation ensues. This routinely involves retrograde travel of blood and floating fragments of endometrial tissue through the uterine tubes onto the ovaries and pelvic peritoneal surfaces in the majority of premenopausal women (78). Although most ovulatory women are presumed to have retrograde menstruation, only a fraction of this population seems to develop endometriosis and its symptoms (1). Quantitatively, obstructive Müllerian abnormalities such as imperforate hymen or transverse vaginal septum strikingly increase retrograde menstrual tissue and very often give rise to endometriosis (79). Premenopausal women with heavy menstrual bleeding also have higher risk of developing endometriosis (80, 81). Qualitatively, endometrial tissue with cancer-driver mutations and epigenetic defects leading to abnormal gene expression is more likely to survive in ectopic locations and give rise to endometriosis (22). All these observations with mechanistic implications, however, likely require the presence of numerous back-to-back ovulatory cycles for endometrial fragments to repeatedly implant and accumulate in an ovarian corpus luteum cyst (ovarian endometrioma), peritoneal surfaces proximal to tubal ostia (peritoneal endometriosis), or the peritoneal pouch between the rectum and the vagina (deep-infiltrating endometriosis) (Figures 2 and 3) (22, 69).

In addition to the retrograde menstruation theory based on ample scientific evidence from human samples, there has been considerable controversy regarding whether other biological processes contribute to endometriosis (15). Based on a report that human donor-derived endometrial cells were detected in eutopic endometrial biopsy samples from human bone marrow recipients, a mouse model was developed to demonstrate that circulating stem cells may contribute to endometriosis (82, 83). Additionally, the peritoneal metaplasia or Müllerian remnant hypothesis was primarily based on some 11 published case reports of male patients, whereby small uterine tissue near the bladder or inguinal canal was discovered because of pain symptoms, frequently stimulated by exogenous estrogen administration to treat prostate cancer (84, 85). The deficiency of or resistance to anti-Müllerian hormone (AMH) during embryonic development most likely caused the persistence of intact uterine tissue, including the myometrium and endometrium, in the pelvis of each of these 46,XY individuals (84, 85). In my opinion, these rare cases of persistent Müllerian tissue in men are not relevant to the hypothesis that peritoneal tissue in women may differentiate to endometriosis via metaplasia or that endometriosis may arise from Müllerian tissue remnants (84, 85).

### Cellular origins

A pathologist typically makes a tissue diagnosis of endometriosis by identifying two of the following three types of cells: (a) endometrial stromal cells, (b) endometrial epithelial cells, and (c) hemosiderin-containing macrophages indicative of chronic bleeding (34). Stromal cells constitute the bulk of endometriotic lesions. Epithelial cells may or may not be present on the surface of an endometriotic lesion and less frequently line the invaginating or glandular portions of these lesions. The origin of the macrophages containing blood pigment in endometriotic tissue is not well understood.

#### Epithelial cells of endometriosis.

Epithelial cells of peritoneal, deep-infiltrating, or ovarian endometriotic lesions share identical cancer-driver mutations with the eutopic endometrial epithelial cells of the same patients (22). At least four different laboratories mapped identical DNA variants or mutations in eutopic endometrium to matched tissues of pelvic endometriosis within the same individuals (21–25, 46, 86). This provided irrefutable evidence that all three forms of pelvic endometriotic lesions originated from the eutopically located endometrium (45).

Interestingly, starting from the teenage years, the endometrium of an average woman progressively accumulates cancer-driver mutations in oligoclones of epithelial cells located in the deepest portions of invaginating mucosal crypts, also called glands (87). Hundreds of cancer-driver genes may be mutated in various endometrial epithelial cell populations of an adult woman (21–25, 46, 86, 87). Given the frequency of this mutational process in the majority of women, it is considered normal (22). What is not well understood is how these mutations contribute to the disease process in a subset of women (44).

In eutopic endometrial tissue, although the most commonly mutated gene is *PIK3CA*, other gain-of-function mutations such as those that affect *KRAS* are also frequently detected (21–25, 46, 86, 87). Although mutations sporadically affect small numbers of cells distributed across the eutopic endometrium, the majority of endometriotic epithelial cells carry these mutations in the same individual (21–25, 46, 86, 87). In particular, epithelial cells with *KRAS* mutations are enriched in endometriotic lesions, suggesting that autonomously activated *KRAS* may confer survival advantages for endometriotic tissue (21, 22, 24, 25, 46, 86). Intriguingly, a number of these mutations in endometriosis also serve as driver mutations for epithelial ovarian cancer (22, 24, 25, 46, 86). Although clear cell and endometrioid ovarian cancers are more strongly associated with an ovarian endometrioma, history of high rates of ovulatory activity is a well-established risk factor for all histological types of epithelial ovarian cancer (44, 88, 89). These data collectively suggest that ovulation followed by retrograde menstruation leads to the seeding of endometrial tissue fragments, including mutated epithelial cells, in an involuting corpus luteum cyst or other inclusion cysts, a small portion of which may eventually evolve into a benign or malignant ovarian neoplasm over decades (44, 69).

Additionally, considerable differences were found between eutopic endometrial and endometriotic epithelial cells with respect to mRNA or protein expression. One of these proteins, 17β-hydroxysteroid dehydrogenase type 2 (HSD17B2), which physiologically catalyzes the enzymatic conversion of estradiol to biologically less potent estrone in eutopic endometrial epithelium during the implantation window, was severely deficient in endometriotic epithelial cells (90–92). The underlying mechanism involves a paracrine relationship between endometrial stromal and epithelial cells (93). It turned out that progesterone via its receptor (PGR) physiologically induces retinol uptake and metabolism in decidualized endometrial stromal cells (94). Consequently, increased levels of retinoic acid, in turn, stimulate HSD17B2 expression and activity in endometrial epithelial cells (93, 95, 96). This paracrine induction of epithelial HSD17B2 expression does not occur in endometriotic tissue because of decreased stromal PGR levels (93–97). Another implantation window marker, glycodelin (PAEP), is expressed at strikingly lower levels in endometriotic epithelial cells compared with the eutopic endometrial tissue (98). PAEP mRNA and protein are significantly lower in eutopic endometrium of women with versus without endometriosis (99). PAEP expression in endometrial tissue was reported to be significantly lower in implantation failure during IVF (100).

#### Stromal cells of endometriosis.

Following the seminal 1994 publication by Ryan et al., which described how to maintain primary endometriotic stromal cells isolated from ovarian endometriomas in culture, a number of groups conducted mechanistic studies on these cells and compared them with eutopic endometrial stromal cells in culture (101, 102). These studies started by demonstrating differential mRNA or protein expression between eutopic and endometriotic stromal cells. They collectively revealed that endometriotic stromal cells bear widespread epigenetic aberrations involving pathological overexpression of transcription factors and nuclear receptors, which transform these cells from a normal endometrial stromal cell to an endometriotic stromal cell (34). Most notably, ectopic expression of NR5A1 (SF-1) and GATA6 together was sufficient to transform a normal endometrial stromal cell to an endometriotic stromal cell that can convert cholesterol to estradiol via activation of the steroidogenic cascade, including aromatase (103). These differences in mRNA or protein expression were also demonstrated in the eutopic endometrium of endometriosis patients, although the difference between eutopic endometrial tissues of women with versus without endometriosis was more subtle compared with expression profiling between endometrium and endometriosis (35, 99, 104). For example, expression of the decidualization markers prolactin and insulin-like growth factor–binding protein-1 (IGFBP1) is reduced in endometriotic stromal cells and in the stromal cells of the endometrium of women with endometriosis compared with disease-free endometrial stromal cells (105).

#### Endometriosis originates from eutopic endometrium of endometriosis patients.

Analyses of epithelial and stromal cells of eutopic endometrium of patients with or without endometriosis and endometriotic lesions with respect to analyses for mutations, DNA methylation, histone modification, and gene expression indicate the following (34): Eutopic endometrium of endometriosis patients harbors oligoclones or localized populations of stromal cells with aberrant mRNA and protein expression and epithelial cells with mutations (22, 25, 104, 106). The great majority of stromal cells in endometriotic peritoneal implants, deep-infiltrating endometriosis, or ovarian endometriomas contain similar aberrant levels of mRNA/protein, most notably NR5A1, GATA6, and aromatase, in part due to DNA methylation differences, which control gene expression (106–108). Likewise, the great majority of epithelial cells in all forms of endometriotic lesions bear an identical driver mutation found in oligoclones of eutopic endometrial cells of the same individual (21–25, 46, 86). The most logical explanation is that if these tissue fragments with molecular aberrations travel to the pelvic cavity via retrograde menstruation, they have a higher potential to implant, survive, and grow in response to estrogen.

### Somatic mutations

Eutopic endometrial tissue of all women accumulates somatic mutations primarily in epithelial cells, starting from the teenage years (87). The mutation burden increases with advancing age (87). In particular, one laboratory reported significant differences between the eutopic endometrial tissues of women with and without adenomyosis or endometriosis with respect to the types of mutations (25). They compared gene mutations in histologically normal-appearing endometrial tissues of patients with adenomyosis, patients with neither adenomyosis nor endometriosis, and patients with endometriosis but without co-occurring adenomyosis (25). In general, the most commonly mutated gene in all three groups was *PIK3CA* (25). Mutation or variant allele frequency in eutopic endometrium was similar with respect to the *PIK3CA* or *PPP2R1A* genes in all three groups. *KRAS* (G12/G13) mutation or variant allele frequency was significantly higher in endometrial tissues of adenomyosis patients compared with disease-free patients (25). In endometrial tissues of patients with endometriosis but without co-occurring adenomyosis, the *KRAS* mutation or variant allele frequency was higher, but the difference did not reach significance (25). Although other studies did not make this direct comparison, their data were also suggestive that the eutopic endometrium of endometriosis patients contained a higher frequency of *KRAS* mutations compared with those of endometriosis-free patients (22, 86). This set of observations raises the question of whether the individuals who develop endometriosis are more likely to have endometrial tissue fragments with *KRAS* mutations in their retrograde menstrual tissue material.

On the other hand, the mutation landscapes of endometriotic or adenomyotic tissues are clearly different from those of eutopic endometrium of the same patient (22, 25, 86). For example, adenomyotic tissue contains almost exclusively *KRAS* mutations (25). Similarly, the majority of epithelial mutations in endometriotic tissue affect the *KRAS* gene (22, 25). Moreover, identical epithelial mutations that affect both eutopic endometrium and endometriosis have been mapped in the matched tissues of each individual patient (22, 25). These observations collectively suggest that endometriosis or adenomyosis originates from eutopic endometrial tissue clones with epithelial mutations that enable these tissues to survive outside of the uterine cavity (21–25, 46, 86).

### Epigenetic defects

#### DNA methylation.

The initial epigenetic studies on endometriosis focused on DNA methylation (108–110). They demonstrated differences between the stromal cells of eutopic endometrium and ovarian endometriomas with respect to the CpG methylation of the promoters or coding regions of distinct transcription factors or nuclear receptors (111). In general, genes such as *NR5A1*, *ESR2* (encoding estrogen receptor-β), and *GATA6* are heavily methylated and suppressed in endometrial stromal cells, whereas they are less methylated and are expressed in endometriotic stromal cells (108–110). In contrast, *GATA2* is methylated and suppressed in endometriosis, whereas it is highly expressed and serves as a critical mediator in progesterone-induced differentiation or decidualization of eutopic endometrial stromal cells (108, 112). In parallel, the failure to downregulate DNA methyltransferase-3B expression in response to physiological differentiating stimuli in endometriotic cells compared with healthy endometrial stroma may contribute to a defective epigenetic fingerprint and gene expression pattern in endometriosis (113). Mechanistically, it is not well understood how individual genes such as *NR5A1*, *ESR2*, *GATA6*, or *GATA2* are differentially methylated in each cell type (108, 112).

Other studies also demonstrated that aberrations in global DNA methylation play a key role in the pathophysiology of endometriosis (114–116). One report suggested that estrogen and progesterone regulate DNA methylome in endometrial cells, and this process is perturbed in endometriosis (115). A recent report suggested that menstrual cycle phase was a major source of DNA methylation of the eutopic endometrium (116). This differential methylation study of endometrium from disease-free or endometriosis patients highlighted candidate genes contributing to disease risk (116).

#### Histone modification.

A limited number of studies have been published regarding the histone modification patterns in endometriosis (117). One study assessed acetylation/methylation levels of histones in whole tissues of endometriosis, eutopic endometrium from endometriosis patients, and endometrium from disease-free individuals (117). Globally, the endometriotic lesions were hypoacetylated at H3 but not H4 compared with disease-free eutopic endometrium (117). Lysine (K)-specific acetylation/methylation levels of histones showed that endometriosis tissue contained significantly lower levels of acetylated H3K9 and H4K16 in comparison with eutopic endometrium from patients or disease-free subjects (117). Both endometriotic and eutopic endometrial tissues from patients were hypermethylated at H3K4, H3K9, and H3K27 compared with normal endometrium of disease-free women. ChIP analysis of endometriotic lesions showed hypoacetylation of H3/H4 around promoter regions of genes previously demonstrated to be downregulated in endometriosis, such as *HOXA10*, *ESR1*, *CDH1*, and *CDKN1A*, compared with disease-free endometrium (117). On the other hand, the *NR5A1* promoter region was enriched with acetylated H3 and H4 in endometriosis versus disease-free tissues, correlating with its reported overexpression in endometriosis (117).

**Figure 4:**
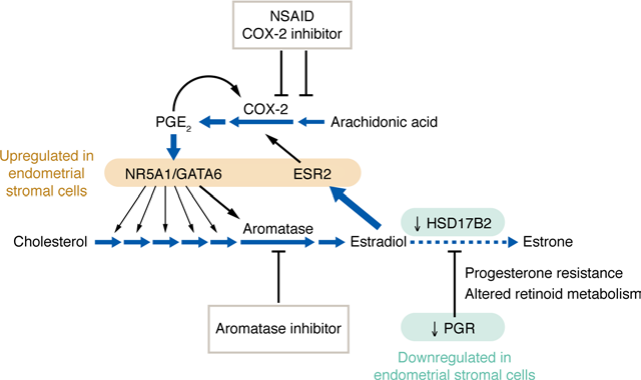
The epigenetic defects that cause gene expression abnormalities in endometriotic cells. The transcription factors NR5A1, GATA6, and ESR2 are pathologically hypomethylated and thus upregulated in the stromal cells of endometriosis, whereas PGR is downregulated in these cells, compared with normal endometrial stromal cells. Consequently, PGE_2_ stimulates the steroidogenic enzymes, including aromatase, to increase the production of estradiol, which stimulates COX-2 to elevate PGE_2_ production. PGE_2_ itself induces COX-2 expression through a short feedback loop. This vicious cycle causes continuous local production of estradiol and PGE_2_ in endometriotic tissue. Low PGR levels in endometriotic stromal cells lead to progesterone resistance and decreased uptake of retinol, leading to decreased retinoic acid production. The enzyme HSD17B2, which is physiologically regulated by retinoic acid in normal endometrium, is downregulated in endometriotic epithelial cells. Deficient HSD17B2 leads to decreased metabolism of estradiol, further contributing to increased estradiol levels in endometriotic tissue.

Epigenetic defects affecting nuclear receptors in endometriotic stromal cells lead to aberrant expression of critical genes. For example, *NR5A1* expression in endometriotic stromal cells activates most of the genes in the steroidogenic cascade, including aromatase, leading to the conversion of cholesterol to estradiol, whereas this is not observed in the eutopic endometrium of disease-free individuals (Figure 4) (102, 103, 107, 118, 119). Excessive *ESR2* expression leads to suppression of *ESR1* (ERα) (120). Ectopic overexpression of *NR5A1* and *GATA6* in a normal endometrial stromal cell is sufficient to confer an endometriotic phenotype with respect to steroidogenesis and failure to differentiate properly (Figure 4) (103, 108). Thus, epigenetic stromal defects in endometriosis profoundly influence the biology of endometriosis.

### Progesterone resistance

Progesterone resistance in endometriotic tissue or eutopic endometrium has been described by numerous laboratories and clinicians in different contexts (35, 91–93, 97, 104, 121–124). It was originally suggested upon the observation that the enzyme HSD17B2, which is involved in inactivation of estradiol, is present in endometrial epithelium but absent in matched endometriotic tissue during the luteal phase, although both tissues are simultaneously exposed to circulating progesterone (Figure 4) (91). Follow-up studies demonstrated that progesterone physiologically acted directly on endometrial stromal cells to alter retinoid uptake and biosynthesis of retinoic acid, which in turn induced epithelial HSD17B2 expression in a paracrine fashion (Figure 4) (93, 96, 125). Pathologically, deficient PGR expression in endometriotic stromal cells disrupted this physiological mechanism and thus the capacity of endometrial epithelial cells to inactivate estradiol during the secretory phase (Figure 4) (93, 94, 96, 125).

A number of other laboratories also reported deficient expression of genes regulated by progesterone in endometriotic tissue or eutopic endometrium of endometriosis patients (99, 104, 126, 127). These included *IGFBP1*, *PAEP*, *HOXA10*, *HOXA11*, *CYP26A1*, *FOXO1A*, *FKBP4*, and *BCL2* (99, 104, 126, 127). *HOXA10* hypermethylation was demonstrated as a mechanism for decreased HOXA10 expression in the endometrium of women with endometriosis (127). Thus, inappropriate methylation of progesterone-regulated genes may be a potential mechanism for progesterone resistance. The underlying mechanisms for the other suppressed genes such as epithelial *PAEP* in tissues of women with endometriosis, however, have remained unclear.

### Prostaglandin production and action

Prostaglandin E_2_ (PGE_2_), a product of the COX/PGES cascade, is the most intensively studied eicosanoid that contributes to inflammatory pain (47). PGE_2_ also plays a central role in the pathophysiology of endometriosis (Figure 4) (48). In endometrium and inflamed tissues such as the peritoneum adjacent to endometriosis, upregulated COX-2 and perhaps also COX-1 produce prostaglandin H_2_ (PGH_2_) from arachidonic acid released by phospholipase A_2_, and then upregulated PGE synthase subtypes produce PGE_2_ from PGH_2_ (Figure 4) (128, 129). PGE_2_ interacts with the G protein–coupled EP_2_ and EP_4_ receptors in the nerve endings or nociceptors, leading to activation of PKA and also EPAC (exchange protein activated by cAMP) to produce PGE_2_-mediated pain (47, 48). In addition to the PGE_2_ receptors at the nerve endings, spinal EP_2_ receptors via PKA activation also contribute to the amplification of PGE_2_-evoked nociceptive input from the spinal cord to higher brain areas where pain is actually perceived (47).

Peritoneal fluid concentrations of PGE_2_ are higher in women with endometriosis, and, in addition to evoking pain, elevated PGE_2_ plays key roles in survival and growth of endometriotic lesions (130, 131). Inhibition of PGE_2_ synthesis suppresses endometriosis and chronic pelvic pain in women and decreases growth of experimental endometriotic lesions in animal models (132, 133). PGE_2_ in endometriotic stromal cells induces the expression of aromatase and other steroidogenic enzymes via the EP_2_/PKA/cAMP pathway and generates estradiol (Figure 4) (102, 103, 119, 134). Estradiol, in turn, stimulates COX-2 expression via ESR2 to enhance PGE_2_ production in uterine cells (Figure 4) (135). Moreover, PGE_2_, via a short feedback loop, induces COX-2 expression via the activation of NF-κB in endometrial stromal cells, leading to a vicious cycle that continuously produces estradiol and PGE_2_ in endometriotic tissue (Figure 4) (102, 129).

Selective inhibition of EP_2_ and EP_4_ decreases survival and growth of human endometriotic cells via altering the expression of a number of gene products, including matrix metalloproteinases (MMPs) and tissue inhibitors of metalloproteinases (TIMPs) (136). Selective inhibition of EP_2_/EP_4_ in an experimental animal model also decreased angiogenesis and innervation of endometriotic lesions, suppressed proinflammatory state of dorsal root ganglia neurons, and significantly reduced proinflammatory, estrogen-dominant, and progesterone-resistant molecular environment in endometriotic tissue (137).

NSAIDs, inhibitors of COX-1 and/or COX-2, suppress endometriosis-associated inflammatory pain by reducing generation of eicosanoids, mainly PGE_2_, while they exhibit gastrointestinal, renal, and cardiovascular toxicities (132, 138). Selective inhibitors of microsomal PGE synthase-1 and subtype-selective antagonists of PGE_2_ receptors, particularly EP_2_ and EP_4_, may be useful as future treatments of endometriosis pain with minimized side effects (137). Thus, PGE_2_ has a substantial impact on endometriosis-induced pain, and pharmacological intervention in PGE_2_ signaling at pelvic peritoneal tissues or central nervous system may serve as a future therapeutic strategy for the treatment of endometriosis-induced pain (47).

## Summary of molecular advances and future therapeutic targets

Pelvic endometriotic tissue evidently originates from shed endometrial fragments that travel in a retrograde manner primarily through the tubes (19, 20, 45). In patients with endometriosis, relatively larger populations of stromal and epithelial cells in the eutopic endometrium display widespread epigenetic abnormalities and mutations that favor increased estradiol and PGE_2_ production and action, progesterone resistance, autonomous activation of KRAS and its downstream survival pathways, and heightened cytokine production and immune cell recruitment (Figure 4). Although these molecular abnormalities have been consistently observed by multiple laboratories, the upstream reproductive process that is indispensable for the establishment of endometriosis and its symptoms remains as repetitious episodes of ovulation followed by retrograde menstruation. That is why the risk of developing endometriosis is extremely low in the absence of ovulatory menses. Ovulation is associated with relatively larger quantities of shed endometrial functionalis tissue that separates from the basalis and travels to the pelvic cavity, increases blood estradiol levels, and directly releases ovulatory fluid with massive amounts of estradiol onto the peritoneal surfaces. The resulting corpus luteum cyst potentially serves as the foundation for a new endometrioma formation. Therefore, the process of ovulation per se strikingly increases the risk for endometriosis in multiple ways.

Eliminating or diminishing ovulation or ovulatory menses in the long term should be the first-line strategy for treating pain caused by endometriosis or potentially preventing endometriosis. Surprisingly, this has not been explicitly addressed by clinicians or researchers. Traditionally, progestins, combination oral contraceptives, or injectable gonadotropin-releasing hormone (GnRH) agonists have been used to suppress ovulatory activity regulated by follicle-stimulating hormone (FSH) and luteinizing hormone (LH) (139–142). More recently, oral GnRH antagonists with estrogen or progestin addback have been introduced (143, 144). The key side effects of GnRH suppression have been hot flashes and decreased bone mineralization (145). New pharmaceutical approaches to suppress ovulation long-term with fewer side effects are urgently needed for prevention and treatment of endometriosis. I speculate that exploiting the inhibin, activin, and follistatin-related pathways or blocking the gonadotropin receptors may produce beneficial future pharmaceutical targets.

## Version 1 07/01/2025

Electronic publication
